# Recent Advances in Electrochemical Sensors for Sulfur-Containing Antioxidants

**DOI:** 10.3390/mi14071440

**Published:** 2023-07-18

**Authors:** Guzel Ziyatdinova, Liliya Gimadutdinova

**Affiliations:** Analytical Chemistry Department, Kazan Federal University, Kremleyevskaya, 18, Kazan 420008, Russia; liliya.gimadutdinova@gmail.com

**Keywords:** voltammetry, amperometry, chemically modified electrodes, sulfur-containing amino acids, antioxidants, bioanalysis, pharmaceutical analysis

## Abstract

Sulfur-containing antioxidants are an important part of the antioxidant defense systems in living organisms under the frame of a thiol–disulfide equilibrium. Among them, l-cysteine, l-homocysteine, l-methionine, glutathione, and α-lipoic acid are the most typical representatives. Their actions in living systems are briefly discussed. Being electroactive, sulfur-containing antioxidants are interesting analytes to be determined using various types of electrochemical sensors. Attention is paid to the chemically modified electrodes with various nanostructured coverages. The analytical capabilities of electrochemical sensors for sulfur-containing antioxidant quantification are summarized and discussed. The data are summarized and presented on the basis of the electrode surface modifier applied, i.e., carbon nanomaterials, metal and metal oxide nanoparticles (NPs) and nanostructures, organic mediators, polymeric coverage, and mixed modifiers. The combination of various types of nanomaterials provides a wider linear dynamic range, lower limits of detection, and higher selectivity in comparison to bare electrodes and sensors based on the one type of surface modifier. The perspective of the combination of chromatography with electrochemical detection providing the possibility for simultaneous determination of sulfur-containing antioxidants in a complex matrix has also been discussed.

## 1. Introduction

Oxidative stress caused by an imbalance in redox status in living organisms is considered one of the main pathways of various pathological states including oncogenesis, diabetes mellitus, neurogenerative diseases, etc. [1,2,3]. The negative effect of oxidative stress is prevented and repaired by the antioxidant defense system consisting of a wide range of antioxidants acting through enzymatic (superoxide dismutase, catalase, glutathione peroxidase, and glutathione reductase) and non-enzymatic (retinol, ascorbic acid, tocopherols, bioflavonoids, thiols, non-enzymatic proteins, etc.) pathways. Among them, sulfur-containing compounds play a significant role in the antioxidant defense system of the living systems via participation in the reactions with reactive oxygen and nitrogen species [4,5,6].

**Scheme 1:**
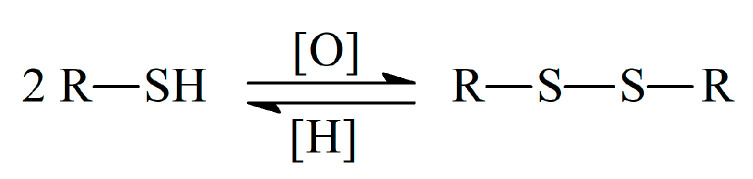
Thiol–disulfide equilibrium.

Sulfur, being an essential element in biology, shows the ability to donate and accept electrons and to form bonds with other S atoms or with carbon, nitrogen, some metals/metalloids, and up to four oxygen atoms [7]. There are three main forms of organic sulfur in living organisms, in particular the sulfhydryl (thiol), thiomethyl, and disulfide groups. The ester or amide bound sulfates are presented in glycosaminoglycans, steroids, and many xenobiotic metabolites [8], but not involved in the antioxidant action. The reactivity of sulfur-containing antioxidants towards free radicals is mainly caused by the presence of the thiol group (–SH). The presence of H-atom in the thiol group provides its reaction with free radicals via H-atom transfer or oxidation to disulfide in reactions with oxidants. The so-called thiol–disulfide equilibrium is formed in living systems (Scheme 1) and is used as one of the oxidative stress indicators [9,10]. The equilibrium is dynamic and its shift is meaningful for the evaluation of the organism redox status [10].

**Scheme 2:**
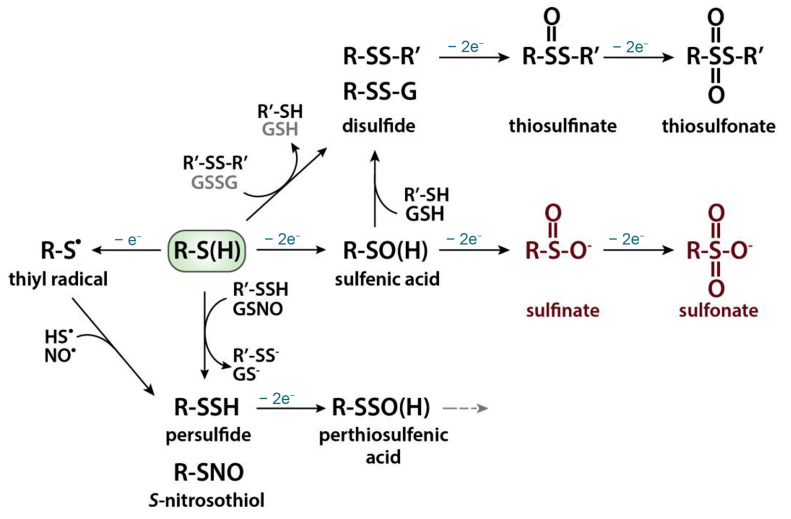
Oxidative thiol modifications. Reprinted from [11], copyright 2019, with permission from Elsevier.

Reactive oxygen/nitrogen species stipulate oxidative modifications of thiols (Scheme 2) [11].

Under exposure to reactive oxygen species, thiols undergo one electron oxidation with formation of thiyl radical or sulfenylation reaction with formation of metastable and highly reactive intermediate sulfenic acid (R–SOH). Nitrosylation reaction occurs in the case of reactive nitrogen species attack with the formation of *S*-nitrosothiol (R–SNO). Sulfenic acid can react with another protein thiol to form an intra- or intermolecular disulfide bond or with a low-molecular weight thiol, such as glutathione (GSH) with the formation of a mixed disulfide (i.e., *S*-glutathionylation). These oxidative thiol modifications are fully reversible, and their reduction is mediated by thiol-based redox systems, most prominently the glutathione/glutathione reductase and thioredoxin/thioredoxin reductase systems. The subsequent oxidation of the sulfenic acid first to sulfinic and then to sulfonic acid is mostly irreversible. The thiyl radical can participate in the *S*-sulfhydration or nitrosylation reaction, giving persulfide or *S*-nitrosothiol, respectively. Further oxidation of a persulfide will generate a perthiosulfenic acid intermediate, which can be further oxidized. Thiolate anions can also directly react with protein disulfides or oxidized low-molecular weight thiols such as oxidized glutathione (GSSG) through thiol–disulfide exchange reactions [11,12]. The oxidative thiol modifications affect enzymatic activities, protein–protein interactions, susceptibility towards other post-translational modifications, protein turnover, and/or sub-cellular localizations [11].

Thus, sulfur-containing antioxidants play an important role in physiological and biochemical processes and are of interest as research objects. Their quantification is required for the evaluation of the redox status of living organisms as well as for diagnosis purposes and pharmaceutical quality control.

The ability of sulfur-containing antioxidants to participate in electron transfer reactions makes possible their electrochemical determination, which is successfully demonstrated using various electrochemical sensors [13,14]. The latter are attractive for practical application due to cost-efficiency, portability, rapidity in combination with high accuracy, sensitivity, and reliability of the target analytes determination.

The current review summarizes recent advances in the electrochemical sensors developed for the quantification of sulfur-containing antioxidants in various matrixes. The main compounds of interest (l-cysteine, l-homocysteine, l-methionine, glutathione, and α-lipoic acid) and their actions in living systems are briefly discussed. The figures of merit for their determination using sensors based on chemically modified electrodes are presented and compared in terms of linear dynamic ranges, limits of detection, and selectivity. Attention is also paid to the analytical capabilities of electrochemical detection in the chromatography of sulfur-containing antioxidants.

## 2. Sulfur-Containing Antioxidants and Their Action in Living Systems

**Figure 1:**
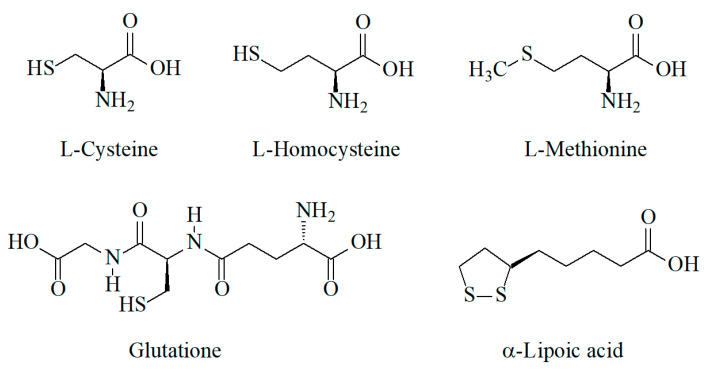
Structure of sulfur-containing antioxidants.

**Figure 2:**
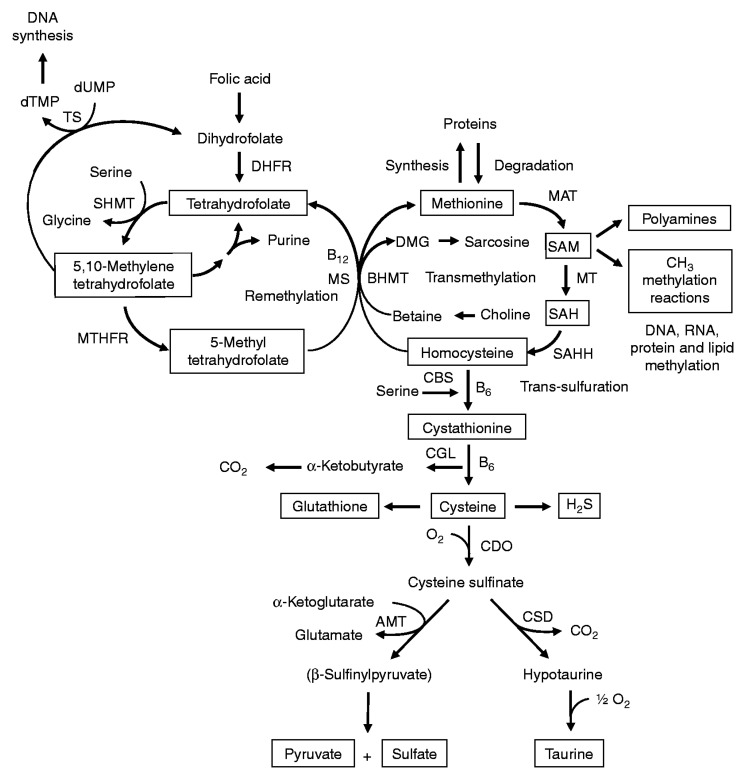
Sulfur-containing amino acid metabolism. AMT—aminotransferase, BHMT—betaine-homocysteine methyltransferase, CBS—cystathionine β-synthase, CDO—cysteine dioxygenase, CGL—cystathionine γ-lyase, CSD—cysteine sulfinate decarboxylase, DHFR—dihydrofolate reductase, DMG—dimethyl-glycine, dTMP—thymidylate, dUMP—deoxyuridylate, MAT—methionine adenosyltransferase, MS—methionine synthase, MT—methyl transferases, MTHFR—methylenetetrahydrofolate reductase, SAH—*S*-adenosylhomocysteine, SAHH—*S*-adenosylhomocysteine hydrolase, SAM—*S*-adenosylmethionine, SHMT—serine hydroxymethyltransferase, TS—thymidylate synthetase. Reprinted from [15], copyright 2009, with permission from Cambridge University Press.

Sulfur-containing antioxidants are synthesized in human cells as well as coming from dietary sources (foodstuff and pharmaceuticals). Amino acids containing thiol (l-cysteine, l-homocysteine) or thiomethyl (methionine) groups, tripeptide glutathione with the thiol group, and α-lipoic acid containing the disulfide group (Figure 1) are the main representatives of these class of antioxidants.

l-methionine is an essential amino acid and a precursor in the biosynthesis of all other S-containing amino acids, in particular l-homocysteine, l-cysteine, and proteins (Figure 2) [15]. On the other hand, the activated form of l-methionine, *S*-adenosylmethionine, serves as a methyl donor in numerous biological reactions [16]. Dietary products (eggs; chicken breasts; some fish; vegetables including spinach, zucchini, mushrooms, and asparagus; dairy; nuts; and beans) are the main sources of l-methionine in living organisms [17]. The normal level of methionine in plasma varies from 13 to 45 μM [18]. High concentrations of methionine provoke oxidative stress that leads to various diseases [17] including neurodegenerative disorders [19,20]. Therefore, the World Health Organization reported the daily consumption of methionine for an adult as 13 mg/kg of body weight [21].

l-homocysteine is a non-essential sulfur-containing amino acid that is derived from methionine metabolism. l-homocysteine is physiologically essential for the cell cycle progression and maintenance of cellular homeostasis [22] Impaired l-homocysteine metabolism leads to its elevated concentration in the blood plasma, i.e., hyperhomocysteinemia. In this case, the overgeneration of free radicals, induced oxidative stress, mitochondrial impairments, systemic inflammation and increased risks of a wide range of eye and heart diseases, neurodegenerative disorders, cancer development and progression, and pregnancy complications occur [22]. The presence of the thiol group in the l-homocysteine molecule possesses its potential antioxidant action under conditions of homeostasis [23]. Total content of l-homocysteine in human plasma has been considered a metabolic disorder parameter in clinical diagnosis.

l-cysteine is a non-essential amino acid, which is produced from methionine in cells via two consequent reactions (transformation of methionine to homocysteine undergoing further transsulfuration [24]), as well as possibly coming from external sources such as foodstuffs and pharmaceuticals. l-cysteine in biological fluids and tissues exists in a free reduced form and two oxidized forms, in particular as a free oxidized form cystine and protein-bound form obtained by *S*-cysteinylation of cysteine residues [25]. l-cysteine participates in the synthesis of proteins as a subunit that possesses folding and stabilization of their tertiary structure, thereby supporting their biological activities [24]. In fact, cysteine plays a key role in development, signal transduction, biological defenses, ageing, and disease. Normal cysteine content in human plasma is in the range of 0.10–0.15 nmol mg^−1^ protein [26]. The deficiency of l-cysteine can lead to the development of various pathologies such as psoriasis, decreased hematopoiesis, oedema, hypnesthesia, hepatic damage, and skin lesions [27]. A high level of l-cystine (the oxidized cysteine form) in plasma gives a negative health effect and is considered a novel pro-oxidative vascular risk factor [28].

Glutathione is a tripeptide consisting of cysteine, glycine, and glutamic acid. It is characterized by high contents (up to 5 mM) in the cells [29]. It is one of the most important natural antioxidants responsible for the prevention of oxidative processes. The glutathione unit of the antioxidant system is involved in the transport of amino acids, defense of cellular macromolecules from endogenous and exogenous reactive oxygen and nitrogen species, peroxides, lipid peroxidation products, and xenobiotics, as well as participating in the regulation of cellular proliferation and apoptosis [30].

α-Lipoic acid (thioctic acid) is a cyclic disulfide and is an important part of antioxidant defense system. It is synthesized in human and other mammalian cells in mitochondria. α-Lipoic acid forms covalent bonds with proteins and plays a vital role in the Krebs cycle [31]. On the other hand, it is widely used as part of dietary supplements and pharmaceutical dosage forms applied in medicine. α-Lipoic acid has multi-beneficial functions in the human organism [32], i.e.,

- Reactive oxygen species scavenging;
- Chelating effect toward transition and heavy metals;
- Oxidative stress harmful effect repair;
- Chemopreventive effect;
- Inhibition of the cell’s apoptosis;
- Antiviral effect;
- Neuro- and cardioprotective activity.

**Scheme 3:**
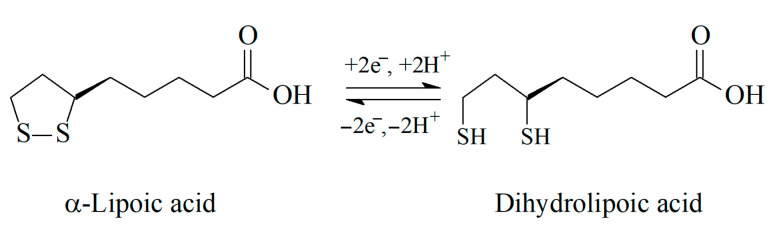
Redox equilibrium of α-lipoic acid.

There is an equilibrium between reduced and oxidized forms of α-lipoic acid in the living organism, in particular between lipoic and dihydrolipoic acids (Scheme 3).

It should be noted that α-lipoic acid is the only antioxidant for which both reduced and oxidized forms exhibit antioxidant activity and protective function, although dihydrolipoic acid is more powerful [33].

Biological aspects of sulfur-containing antioxidant actions, their metabolism, and implementation in diseases pathogenesis are discussed in detail in reviews [10,16,19,22,24,25,30,31,32].

Thus, information regarding sulfur-containing antioxidants contents in biological fluids and tissues is required for fundamental studies in biochemistry and pharmacology and in practical medicine for the adequate diagnosis and treatment of various diseases.

## 3. Electrochemical Sensors for S-Containing Antioxidants

### 3.1. Bare Electrodes

Electrochemical sensors have been shown to be an effective tool in antioxidants’ electroanalysis [34]. l-cysteine, l-homocysteine, l-methionine, and glutathione show lack of electroactivity at the traditional carbon-based electrodes [35,36]. l-cysteine and l-homocysteine are oxidized at high anodic potentials and the oxidation currents are extremely low despite the high concentration studied. l-methionine and glutathione are silent on the voltammograms in the reasonable electrochemical window, in this case [36]. The successful example of l-methionine determination has been shown using screen-printed graphite macroelectrode, although the analytical characteristics obtained (linear dynamic range of 500–5000 μM with the detection limit of 95 μM) are not impressive [37] as they do not cover physiological concentrations. The voltammetric determination of glutathione has been realized on the platinum electrode in 0.05 M H_2_SO_4_ using linear sweep voltammetry (LSV) within the 91.5–2140 μM concentration range with a detection limit of 19 μM [38]. This approach has been applied to whole blood sample analyses after preliminary precipitation and isolation of glutathione using cadmium sulfate.

α-Lipoic acid shows higher electrochemical activity and can be determined using platinum [39], glassy carbon (GCE) [40,41], carbon fiber microelectrode [42,43] or boron-doped diamond [44,45] electrodes. The linear dynamic ranges are mainly from dozens to hundreds of μM excluding boron-doped diamond electrodes, for which a 0.3–105 μM linear dynamic range with a detection limit of 0.088 in Britton–Robinson buffer pH 3.0 [44] and 0.0582–400 μM with a detection limit of 0.0194 μM in McIlvaine buffer pH 3.0 [45] have been achieved in differential pulse voltammetry (DPV).

In summary, bare electrodes show insufficient analytical characteristics of sulfur-containing antioxidants. Several of them do not show electrochemical activity at the bare electrode. Another important drawback of traditional electrodes is fouling of the surface and low selectivity toward sulfur-containing compounds in the presence of structural analogues. Therefore, modification of the electrode surface is required to solve this problem.

### 3.2. Sensors Based on Modified Electrodes

**Figure 3:**
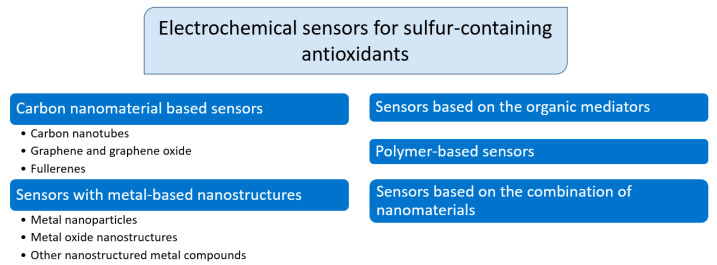
Classification of electrochemical sensors based on chemically modified electrodes for sulfur-containing antioxidants.

The great variety of electrode materials gives the opportunity to control the characteristics of sulfur-containing antioxidants. This research field was investigated more with the development of chemically modified electrodes based on various nanosized materials (Figure 3).

The functionalization and combination of modifiers of different types have been widely used in order to obtain their synergetic effect on the electrooxidation of sulfur-containing antioxidants. Achievements in electrochemical sensors for sulfur-containing antioxidants are discussed below.

#### 3.2.1. Carbon Nanomaterial-Based Sensors

Carbon nanomaterials have become one of the most popular materials for the creation of electrochemical sensors [46]. Among them, multi-walled carbon nanotubes (MWCNTs), carbon nanofibers, graphene, and graphene oxide (GO), as well as fullerenes, have been used as electrode surface modifiers. Analytical capabilities of carbon nanomaterials based electrochemical sensors in the analysis of sulfur-containing antioxidants are presented in Table 1.

**Figure 4:**
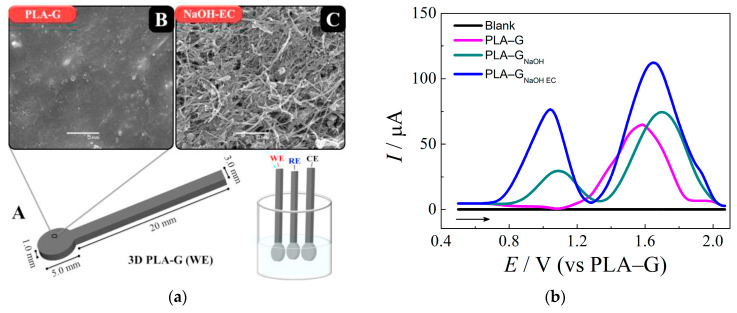
Graphene 3D-printed electrode based on the polylactic acid/graphene (PLA–G) for l-methionine: (**a**) electrode dimensions and electrochemical cell composed for three 3D-printed electrodes (**A**), SEM images of the electrodes surfaces before (**B**) and after chemical and electrochemical activation (**C**); (**b**) baseline-corrected square wave voltammograms obtained for l-methionine at untreated and treated 3D-printed electrodes in 0.10 M phosphate buffer pH 7.4. Reprinted from [48], copyright 2020, with permission from Elsevier.

An original voltammetric approach for l-methionine determination has been developed using simple and low-cost 3D-printed electrodes based on polylactic acid/graphene filament [48]. The electrodes obtained by standard 3D printing have undergone a two-step activation. First, chemical activation via immersion in the 3.0 M NaOH for 30 min has been performed. Then, electrochemical activation by potentiostatic treatment at 1.8 V for 900 s in 0.10 M phosphate buffer pH 7.4, followed by a one cyclic voltammetric scan from 0.0 to −1.8 V at a potential scan rate of 50 mV s^−1^, has been carried out. This treatment leads to dramatic changes in the electrode surface morphology and the response of l-methionine (Figure 4).

Electrode activation provides higher anodic peak currents compared to bare printed electrodes and electrodes after chemical treatment (Figure 4b). Moreover, anodic peaks are shifted to less positive potentials confirming an increase in the electron transfer rate in spite of the pseudo-reference electrode usage. There is an absence of an interference effect from ascorbic acid, dopamine, l-alanine, and uric acid, while glucose and cysteine affect the oxidation peaks of methionine. The applicability of sensors to the analysis of human serum has been shown by a spiked procedure [48].

In general, the structural and size effects of carbon nanomaterials change the electrode surface properties, in particular its conductivity and area, including on the electroactive one. The structural defects of carbon nanomaterials (a large edge plane/basal plane ratio, and presence of oxygen-containing functional groups) would induce local electron disturbance or facilitate the formation of active sites. Therefore, a high rate of electron transfer reactions is provided at the modified electrode. The oxidation potential of l-cysteine is shifted to less positive values, while l-methionine and glutathione become electroactive in the reasonable electrochemical window [36]. The higher oxidation currents of the analytes are caused by an increase in the electrode electroactive surface area that is results in a partial improvement in the analytical characteristics of sulfur-containing antioxidants compared to bare GCE and platinum electrodes. Nevertheless, even the application of pulse modes of voltammetry [47,48,53] and amperometry [50,51,52] does not show significant improvement in the sulfur-containing antioxidant response, which is probably caused by low affinity of the thiol group towards carbon nanomaterials. Another aspect that is out of consideration in the majority of cases is the selectivity of the electrode response to the target S-containing compound. This is also reflected in the lack of practical applications of the sensors developed excluding analysis of pharmaceutical dosage forms.

Thus, the use of carbon nanomaterials as a sensing layer is insufficient for the sensitive and selective determination of sulfur-containing antioxidants. The application area is limited to pharmaceutical dosage forms with a high content of analytes.

#### 3.2.2. Electrochemical Sensors Based on Metal and Metal Oxide Nanostructures

Metal and metal oxide nanostructures, in particular nanoparticles (NPs), are the most widely used type of electrode surface modifiers in the electroanalysis of sulfur-containing antioxidants. Other inorganic nanostructured compounds (sulfides, hydroxides, metal-doped nanomaterials, etc.) have been successfully synthetized and used as electrocatalysts and sensing layers for sulfur-containing antioxidants. The corresponding data are presented in Table 2.

**Figure 5:**
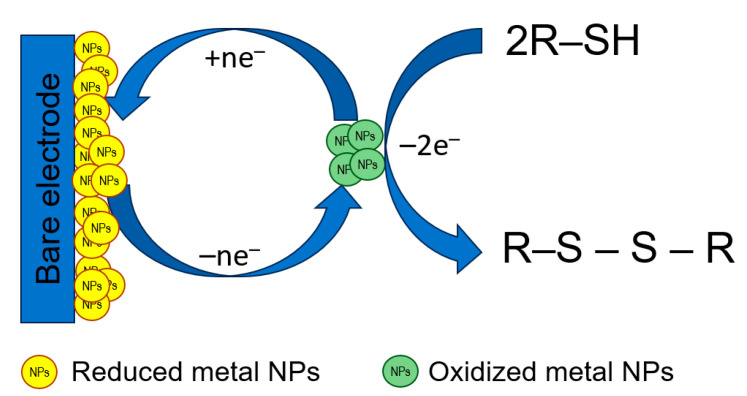
The principal scheme of electrocatalysis at the electrodes modified with metal NPs.

Metal-based NPs act as electrocatalysts toward S-containing antioxidants improving the electron transfer rate as well as increasing the number of electroactive centers at the electrode surface. The principal scheme of electrocatalysis at the electrodes modified with metal NPs is shown in Figure 5.

Noble metals such as Au and Ag, as well as Cu, have been shown to be an effective electrocatalysts for the oxidation of compounds containing a free thiol group (l-cysteine, l-homocysteine, and glutathione) that are also caused by the high affinity of sulfur to these metals [94]. Currently, the application of metal NPs as a single modifier and sensing layer is almost outside of the research paradigm because their combinations with other types of modifiers are more effective in electroanalysis of sulfur-containing antioxidants (see Section 3.2.5.).

Recently, bimetallic NPs have received attention as a more effective modifier due to the synergetic effect of each metal [63,64,65,66,67,95]. The electrocatalytic performance parameters can be varied through controlling the size, composition, morphology, and crystal structure of the nanoparticles [95]. There are two general approaches to the synthesis of bimetallic NPs, i.e., chemical synthesis by various methods using, as a rule, many additional reagents which have to be removed after and electrochemical deposition in potentiostatic or galvanostatic modes which are much faster, easier in realization and do not require additional reagents and toxic solvents. Furthermore, the bimetallic NPs are formed directly at the working electrode surface, which excludes the necessity to remove support materials.

The modification has been performed by drop casting of the bimetal colloidal water–ethanol solution at the electrode surface [63,65]. Then, 10 μL of 5% Nafion-117 has been placed at the modified electrode to provide a membrane facilitating bimetallic NPs sticking to the electrode surface and working as a proton exchange layer. The opposite strategy to electrode surface modification has been applied in [67]. The copper electrode has been covered with 1% Nafion layer. Then, it was immersed in a standard solution of 0.85 mg·L^−1^ of Ag^+^ and 0.15 mg·L^−1^ of Hg^2+^ or Bi^3+^ for 3 h. After the removal of non-adsorbed metal ions, controlled potential coulometry at −1.2 V for 300 s in a solution containing 1 M KNO_3_ and 0.1 M HNO_3_ has been applied to obtain bimetallic NPs-modified electrodes. This approach to electrode modification is simpler and more express than chemical production of metal NPs. Furthermore, the reproducibility of the nanomaterials’ characteristics and electrode surface coverage is better.

Fabrication of a Ru–Pt bimetallic monolayer at the surface of the nanoporous gold film electrode has been carried out by underpotential deposition of copper, the layer of which has been spontaneously replaced by the reduction in Pt(IV) and Ru(III) ions from their aqueous solution containing 5 mM Pt(IV) and 20 mM Ru(III). The reaction takes 10 min at open circuit potential. Such an approach allows obtaining of a mono- or sub-monolayer of Pt–Ru that acts as a bimetallic nanostructure and provides a high electrocatalytic effect toward l-methionine oxidation [64].

Analytical characteristics of sulfur-containing antioxidants obtained by sensors based on metal and bimetallic NPs and nanostructures are comparable to those at the carbon nanomaterial-based electrodes. The selectivity of sensor response is partially improved but only inorganic ions, glucose, ascorbic and uric acids are studied as potential interferences. Simultaneous determination of sulfur-containing antioxidants is impossible due to the close oxidation potentials. Homocysteine is fully excluded from the studies using metal NPs-based electrodes.

**Figure 6:**
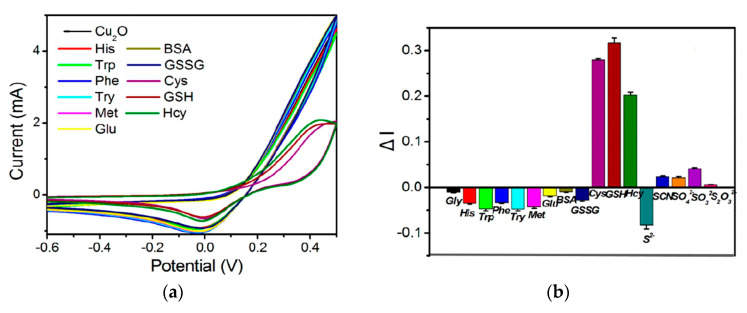
Response of various bioactive compounds at the fluorine-doped tin oxide glass electrode modified with the electrodeposited Cu_2_O nanostructures: (**a**) cyclic voltammograms of the electrode in the presence of 11 biomolecules in the 0.5 M Na_2_SO_4_ (pH 7.1) with a potential scan rate of 100 mV s^–1^; (**b**) the change in reduction current peak of Cu_2_O in the presence of biomolecules and anions (20 μM). Reprinted from [76], copyright 2021, with permission from Elsevier.

Metal oxide nanostructures have been widely developed as a sensing material in electroanalysis of sulfur-containing antioxidants. Electroactive metal oxides like CuO, Cu_2_O, NiO, etc. act on the principles of electrocatalysis similar to metal NPs. The thiol group of sulfur-containing antioxidants can easily interact with Cu_2_O to form a Cu-S bond by transferring the lone pair of electrons from the sulfur atom to the vacant orbitals of Cu(I) [76]. Therefore, the addition of cysteine, homocysteine, or glutathione noticeably alters the Cu_2_O reduction peak current (Figure 6). Other amino acids and inorganic ions do not show an interference effect.

**Figure 7:**
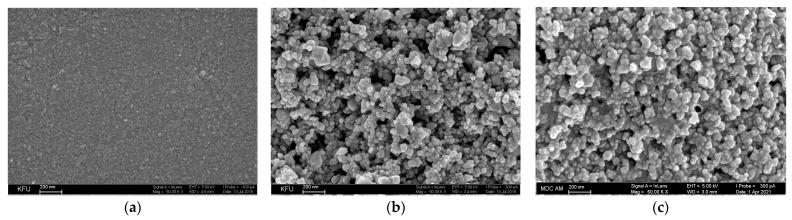
Surface morphology of the electrodes obtained by SEM: (**a**) bare GCE; (**b**) SnO_2_ NPs in cetyltriphenylphosphonium bromide/GCE; reprinted from [77], copyright 2019, with permission from Springer Nature; (**c**) CeO_2_·Fe_2_O_3_ NPs/GCE; reprinted from [78]; published under Creative Commons CC BY license.

Electrochemically inert oxides such as CeO_2_, SnO_2_, and their composites provide high roughness and porosity of the electrode surface area (Figure 7).

Modification of the electrode surface also leads to an increase in its electroactive area and the possibility of the analytes adsorption that leads to an increase in sulfur-containing antioxidant oxidation currents and, consequently, sensitivity of determination. This has been successfully demonstrated on an example of α-lipoic acid [77,78]. Application of CeO_2_·Fe_2_O_3_ NPs as a sensing layer provides the best analytical characteristics of α-lipoic acid among those reported to date [78]. Furthermore, the high selectivity of the sensor response in the presence of l-cysteine (up to the 5 µM level), methionine, and l-cystine has been achieved.

Doping of metal to transition metal oxide nanostructures or vice versa allows an increase in the conductivity of the sensing layer and favorable change to the sensor response to sulfur-containing antioxidants [82,83]. Sb has been shown as a promising p-type dopant to enhance ZnO properties [82]. This leads to changes in the elbow-shaped Sb-doped ZnO nanowire electrode surface morphology and significant improvement in the l-cysteine response due to the increased number of defects in Sb-doped ZnO nanowires. An electrodeposited Co-Gd_2_O_3_ nanocomposite with embedded Gd_2_O_3_ NPs provides high conductivity, high surface area and increases the number of active sites, which favors cysteine adsorption [83]. The formation of the electrocatalytical CoOOH/CoO_2_ species accelerates the electron transfer, which increases the sensitivity of Co-Gd_2_O_3_ nanocomposite vs. bare cobalt.

**Figure 8:**
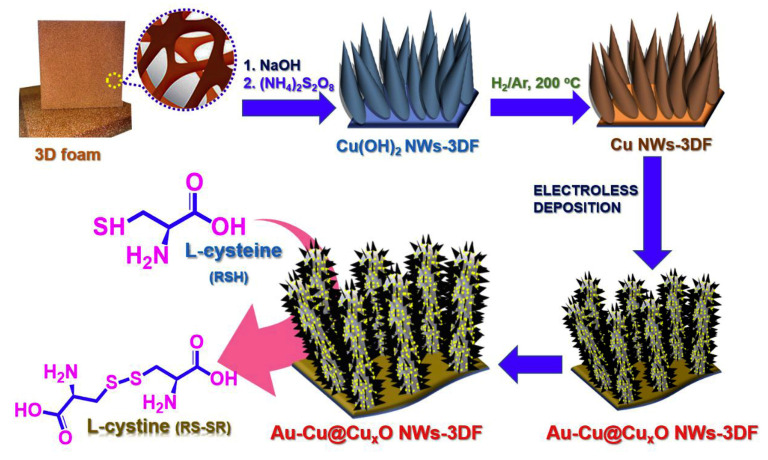
Schematic illustration for fabrication of Au NPs–Cu@Cu_x_O nanowire 3D foam hybrid. Reprinted from [88], copyright 2019, with permission from Elsevier.

A Au NPs–Cu@Cu_x_O nanowire 3D foam hybrid is another type of nanocomposite acting as an electrode material [88]. The synthesis is presented in Figure 8. A strong interaction between Au and Cu phases provides synergistic effects, which improves the exposed electroactive site number, accelerate the charge transfer, and increases the surface area. The hybrid has been used as a novel binder-free self-supported sensor towards l-cysteine. The amperometric response of 1 mM l-cysteine is selective in the presence of 0.05 mM dopamine, KCl, urea, ascorbic and uric acids, 0.5 mM glucose, glycine, H_2_O_2_, and citric and oxalic acid. Another meaningful advantage of the developed sensor is high storage stability (l-cysteine current remains 94.6% after four weeks of storage), making it applicable in real practice.

**Figure 9:**
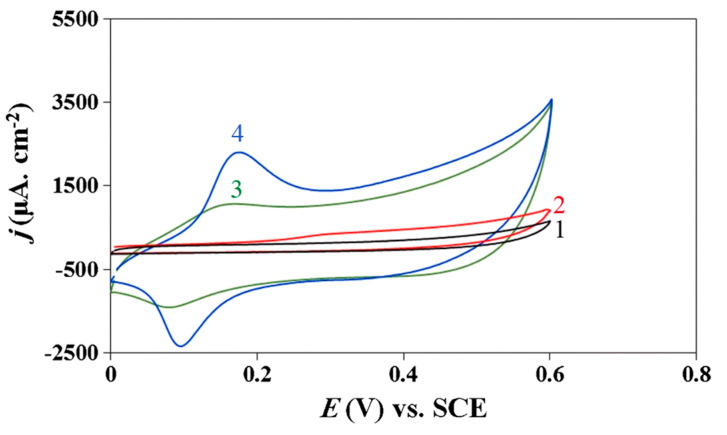
Cyclic voltammograms of bare PGE (1, 2) and Co(II)/Al(III) layered double hydroxide/PGE (3, 4) in 0.1 M NaOH (curves 1 and 3) and in the presence of 1 mM l-cysteine (curves 2 and 4); potential scan rate is 100 mV s^–1^. Reprinted from [87], copyright 2019, with permission from Springer Nature.

Other nanomaterials of metal-containing compounds have been recently developed and tested as a sensing platform for sulfur-containing antioxidants, mainly l-cysteine. The analytical characteristics achieved are similar to metals and metal NP-based sensors excluding Co(II)/Al(III) layered double hydroxide/PGE for l-cysteine [87]; in situ electrodeposited Ag NPs/NiFe contained Prussian blue analog/GCE for glutathione [92] and ZnS/ZnAl_2_S_4_ nanocomposite-CPE for l-methionine [93], for which impressive low detection limits and wide linear dynamic ranges including low concentration levels have been obtained. Layered double hydroxides, namely anion exchangers, have attracted attention due to adjustable composition, easiness of preparation, low cost, anion exchange properties, good catalytic activity, thermal stabilities, large surface-to-volume ratio, and strong adsorption ability [87]. Cobalt oxidation and reduction peaks in the double hydroxides layer in basic medium are significantly increased in the presence of l-cysteine (Figure 9). The disadvantage of the approach is that an alkaline medium is required for this determination. Nevertheless, this is a typical problem in the case of metal and metal oxide NP-modified electrodes.

**Figure 10:**
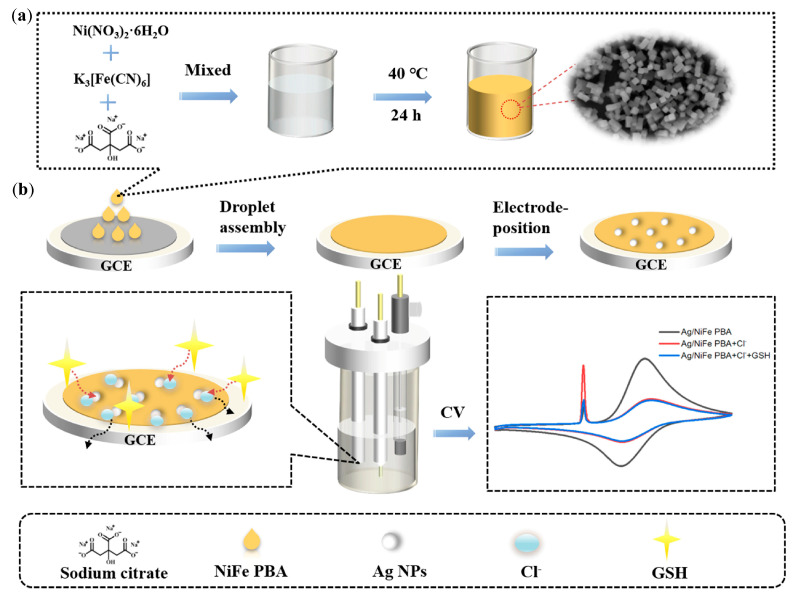
Schematic illustration of the in situ electrodeposited Ag nanoparticles modified with nickel and iron contained in Prussian-blue-analog-based GCE: (**a**) synthesis procedures of NiFe PBA; (**b**) the determination of glutathione via the solid-state electrochemical property of AgCl based on Ag/NiFe PBA/GCE. Reprinted from [92], copyright 2022, with permission from Elsevier.

An interesting highly sensitive dual-signal intrinsic self-calibration electrochemical sensor for glutathione has been fabricated using in situ electrodeposited silver nanoparticles modified with Prussian blue analog containing nickel and iron (Ag/NiFe PBA) (Figure 10) [92].

Ag NPs/NiFe PBA modified electrodes show two well-resolved oxidation peaks ascribed to species of Ag and Fe. A sharp characteristic peak with a largely enhanced current intensity appears in the presence of chloride ions caused by the solid-state electrochemistry of silver chloride (AgCl). A sensitive decrease in the peak current of AgCl has been obtained after the addition of glutathione due to the specific interaction of Ag–glutathione accompanied by the “crowding-out effect”. At the same time, the peak current of NiFe PBA remains unchanged. Therefore, the ratio of the peak currents (*I*_AgCl_/*I*_Fe_) has been used as an analytical signal. The superior performance (an extremely low detection limit and the widest linear dynamic range) compared to other electrochemical methods has been achieved. Another significant advantage of the developed sensor is a negligible response of other sulfur-containing compounds (homocysteine, methionine and thiophenol, cysteine, oxidized glutathione, and so on) with a five-fold excess confirming excellent selectivity for glutathione. Such behavior is attributed to the specific recognition and competitive reaction of the Ag–glutathione complex with AgCl [92]. The data obtained makes a ratiometric sensor based on the solid-state electrochemistry of a AgCl-promoted signal amplification strategy a perspective tool for application in bio- and food analysis.

Electrochemical sensors based on metal and metal oxide nanostructures show relatively similar analytical characteristics. The variation in the modifiers just enlarges the number of available nanomaterials differing in conductivity and morphology, production costs, and easiness of synthesis, but does not provide a significant improvement to the figure of merit of the sensors, excluding several cases mentioned above [77,78,87,92,93]. l-cysteine is the most often studied analyte, while l-homocysteine is out of investigation.

#### 3.2.3. Sensors Based on Organic Mediators

Electrocatalytic effects towards electrode reactions of sulfur-containing antioxidants have been observed using various types of organic compounds undergoing a reversible redox behavior at the suitable electrode surface. The most commonly used redox mediators are cobalt phthalocyanine [96,97], ferrocene, and its derivatives [98,99].

Batch injection analysis with amperometric detection using a pyrolytic graphite electrode modified with cobalt phthalocyanine has been developed for the determination of α-lipoic acid [96]. The electrode surface has been modified using an irreversible adsorption technique. α-Lipoic acid oxidation currents in neutral medium under conditions of batch injection analysis are significantly increased vs. bare electrodes and demonstrate good reproducibility and stability. The fast washout process (<20 s) provides a high throughput for the system (at least 180 samples per hour). The linear dynamic range of 1.3–100 μM with a detection limit of 0.015 μM has been obtained. The approach has been successfully applied in the analysis of dietary pharmaceutical supplements and synthetic urine.

**Figure 11:**
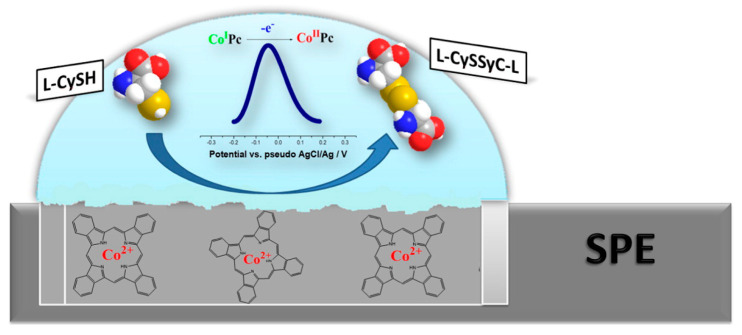
Schematic illustration of Co(II)-phthalocyanine-based disposable screen-printed electrode action towards l-cysteine. Reprinted from [97], copyright 2016, with permission from Elsevier.

A disposable screen-printed electrode modified with Co(II)-phthalocyanine has demonstrated excellent electrocatalytic activity towards the electrochemical oxidation of l-cysteine [97]. A mediator has been added to the carbon-graphite ink formulation prior to the printing procedure. l-cysteine oxidation occurs at a very low potential of −0.04 V (Figure 11) allowing exclusion of the interference effects of many bioactive compounds typical for real samples.

Under conditions of square-wave voltammetry in 0.1 M phosphate buffer pH 7.0, the linear dynamic range is 2.6–200 μM l-cysteine with detection and quantification limits of 3.8 and 14.0 μM, respectively [97]. The absence of the interference effects from of l-tryptophan, l-tyrosine, l-serine, l-asparagine, l-glutamine, l-glutamic acid, l-alanine, l-proline, l-methionine, l-aspartic acid, l-histidine and l-phenylalanine has been shown. The sensor has been applied in the analysis of embryo cell culture media.

Ferrocene derivatives have been used in combination with MWCNTs as constituents of carbon paste electrodes [98,99]. *N*-4,4′-azodianiline(ferrocenyl Schiff base) has been synthetized and successfully used as electrocatalyst towards glutathione oxidation [98]. A differential pulse voltammetric response at 0.54 V in phosphate buffer pH 6.0 has been used for the quantification of glutathione in the range of 0.3–130 and 130–3350 μM with a detection limit of 0.08 μM. The selectivity of glutathione response has been shown in the presence of a 600-fold excess of glucose; a 500-fold excess of fructose, sucrose, lactose, urea, and aspartic acid; a 200-fold excess of citrate; and a 20-fold excess of cysteine and ascorbic acid.

**Scheme 4:**
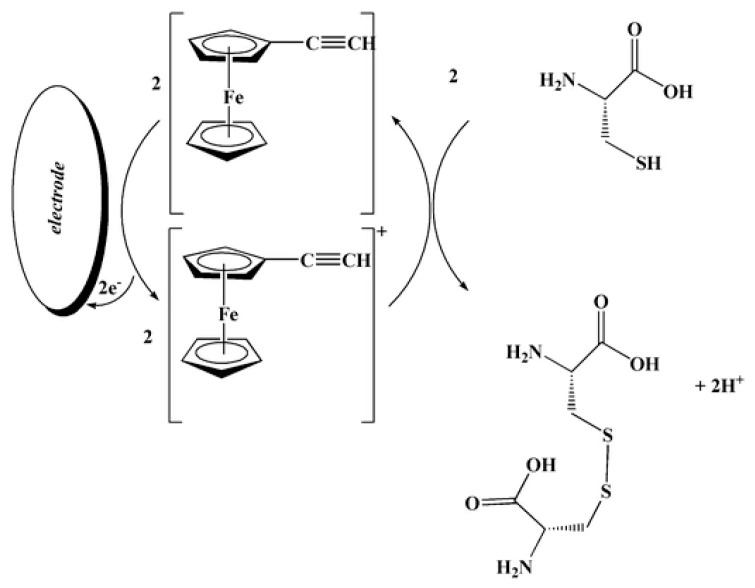
Suggested response mechanism of the ethynylferrocene-based sensor for l-cysteine. Reprinted from [97], copyright 2012, with permission from Springer Nature.

An ethynylferrocene-modified MWCNT paste electrode has been developed for the voltammetric determination of l-cysteine in the presence of folic acid [99]. The peak potential of l-cysteine oxidation at this electrode is shifted by about 380 and 410 mV towards less positive values compared with that of a MWCNTs-modified paste electrode and bare carbon paste electrode, and oxidation currents are significantly increased. Such behavior indicated an electrocatalytic effect of the ethynylferrocene. The electrocatalysis scheme is presented below (Scheme 4).

The square-wave voltammetric response of l-cysteine is linear in the concentration range of 0.2–250 μM. The detection limit of 0.07 μM has been obtained. The selectivity of the sensor towards l-cysteine has been observed in the presence of tryptophan, leucine, l-threonine, l-phenylalanine, glycine, methionine, alanine, valine, urea, thiourea, uric acid, glucose, sucrose, lactose, fructose, inorganic ions, and starch. Sensor practical applicability has been tested on urine, serum, and water samples [99].

Further development in the field is focused on the application of other phenolic compounds as a redox mediators [100,101,102,103,104,105]. Successful incorporation of *p*-aminophenol into multi-walled carbon nanotube paste matrixes allowed fabrication of a paste electrode giving high electrocatalytic activity for glutathione oxidation, producing a sharp oxidation peak current at about +0.285 vs. a Ag/AgCl reference electrode at pH 5.0 [100]. A linear dynamic range of 0.20–100 μM glutathione with a detection limit of 0.090 μM has been obtained using square-wave voltammetry. The approach is shown as selective, simple, and precise, allowing determination of glutathione in hemolyzed erythrocytes, tablets, and urine samples.

Quite similar approaches have been applied for the voltammetric quantification of homocysteine using MWCNT paste electrodes modified with chlorpromazine [101] or isoprenaline [102] as electron transfer mediators. Square-wave voltammetry shows a wide 0.1–210 μM linear dynamic range with a detection limit of 0.08 μM at pH 4.0 [101], while linear sweep voltammetry has a range of 5.0 to 800 μM with a detection limit of 3.3 μM at pH 3.5 [102]. Both methods have been tested on spiked human urine and serum with satisfactory results. The level of l-homocysteine in the samples cannot be determined using sensors developed as far as it is below the detection limits.

**Figure 12:**
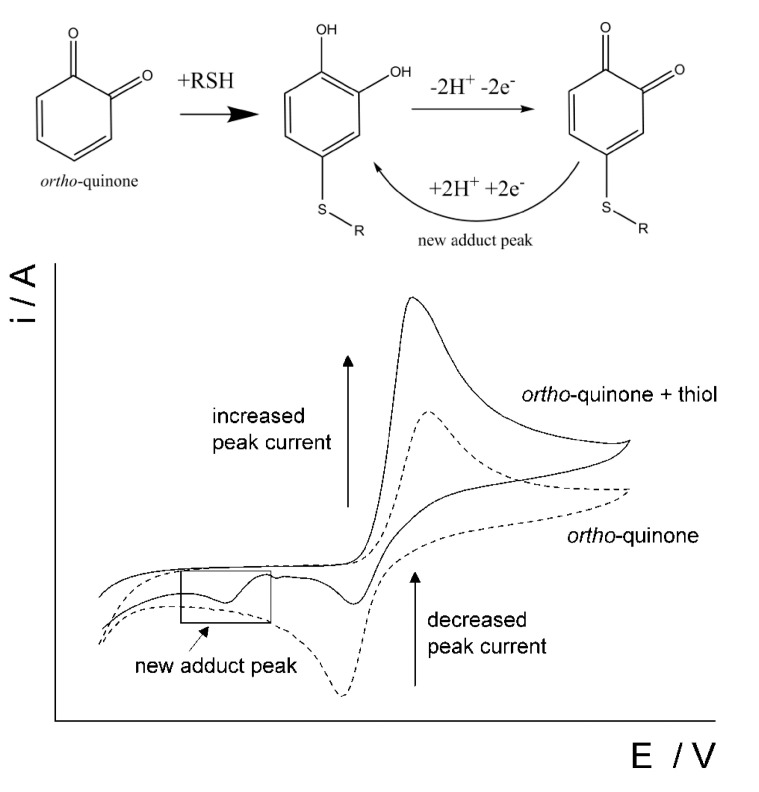
Schematic representation of the reaction between a thiol and ortho-quinone and the resulting voltammetric changes. Reprinted from [103], published under Creative Commons CC BY license.

Several studies have been focused on the development of voltammetric sensors for the l-homocysteine and its simultaneous determination in the presence of other sulfur-containing antioxidants, in particular l-cysteine and glutathione [103,104,105]. The original approach is based on the interaction of thiols with the *o*-quinone via a 1,4-Michael addition reaction with thiols (scheme in Figure 12). *o*-Quinone is electrochemically generated from catechol and the *o*-quinone is susceptible to a nucleophilic attack by a thiol. As evidence of the 1,4-Michael addition reaction, the appearance of a new adduct peak coinciding with an increase in the forward peak and a decrease in the back peak is typically seen in voltammetry (Figure 12) [103,104,105]. The new adduct peak is caused by a reduction in the adduct formed by glutathione or homocysteine, while cysteine does not typically produce a new signal.

This approach has been successfully realized on screen-printed electrodes and carbon nanotube-modified screen-printed electrodes [103], bare GCE and carbon nanotube-modified GCE [104], and MWCNT-modified electrodes [105]. The analytical characteristics obtained allow practical application of the sensors for real samples analysis.

The same principle of a 1,4-Michael addition reaction of *o*-quinone with thiols is used with another primary compound, in particular 3,5-di-*tert*-buthylcatechol [106] or cyclotricatechylene [107], for the determination of homocysteine in the presence of cysteine and glutathione, or cysteine in the presence of homocysteine and glutathione, respectively. In the case of cyclotricatechylene, a carbon electrode was modified with cyclotriveratrylene, which undergoes further electrochemical oxidation. The analytical characteristics of the analytes are close to previous ones using catechol as a reaction promoter.

Quantification of reduced glutathione has been achieved in the presence of l-cysteine and cysteamine hydrochloride using the voltammetric response of the product of reaction between glutathione and catechol acting as the electron mediator on single-walled carbon nanotube-modified GCEs [108]. The adduct formed undergoes polymerization at the electrode surface. The oxidation peak at +0.160 V has been used for analytical purposes. The concentration range to be quantified is 1.0–500.0 μM with a detection limit of 0.5 μM of glutathione. Analysis of the leakage of intracellular glutathione content in HeLa cells following doxorubicin-induced cell apoptosis has been successfully performed using a developed approach.

**Figure 13:**
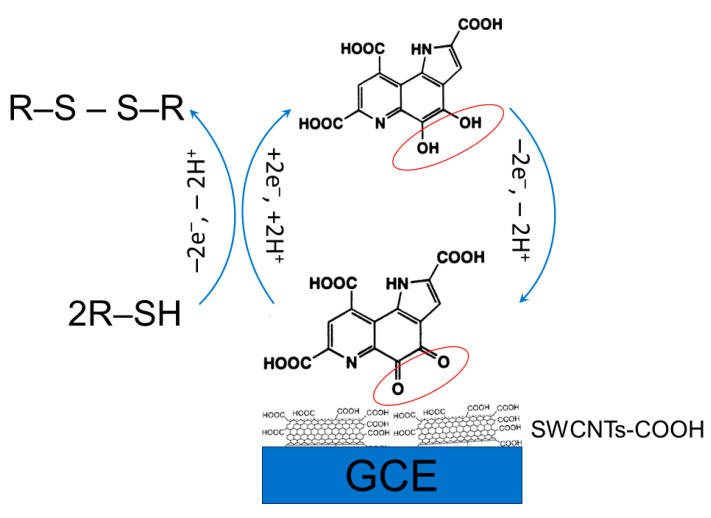
Schematic representation of the electrocatalytic response of a pyrroloquinoline quinone modified electrode.

The electrocatalytic effect of pyrroloquinoline quinone immobilized on the single-walled carbon nanotube-modified electrode towards thiols has been shown in phosphate buffer pH 7.5 [109]. The pyrroloquinoline quinone undergoes a reversible electron transfer at the electrode modified with single-walled carbon nanotubes with the formation of a redox pair at −0.085 and −0.125 V, respectively. Single-walled carbon nanotubes act as efficient conductive pathways between the pyrroloquinoline quinone and glassy carbon electrode. Immobilized pyrroloquinoline quinone chemically oxidizes S-containing antioxidants to disulfides while forming a reduced form. The mechanism of the electrode response is presented in Figure 13.

Oxidation potentials of l-cysteine, l-homocysteine, *N*-acetyl-l-cysteine, and glutathione are in the rage of 0.272–0.321 V. Application of pyrroloquinoline quinone provides a significant improvement to the electrode sensitivity and enlargement of linear dynamic ranges, which are 1.0–2000, 5–5000, 5–5000, and 30–3000 μM for l-cysteine, l-homocysteine, *N*-acetyl-l-cysteine, and glutathione, respectively, under conditions of amperometry. The detection limits of 0.7, 2.2, 3.2, and 6.2, respectively, have been obtained [109].

**Figure 14:**
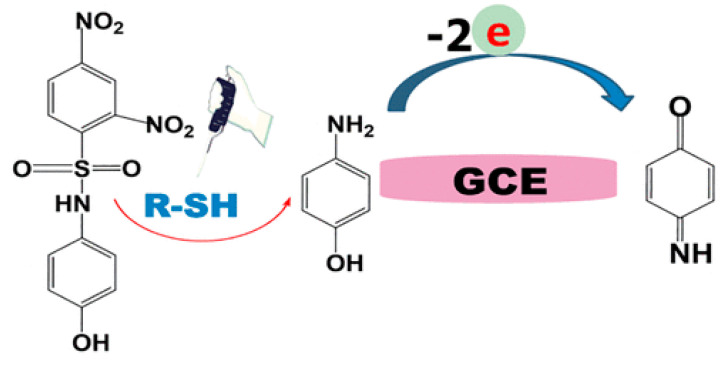
Schematic illustration of 2,4-dinitrobenzenesulfonyl-containing probe reaction with thiols and further electrochemical transformation of *p*-aminophenol. Reprinted from [110], copyright 2022, with permission from the American Chemical Society.

Another indirect voltammetric method for biothiol determination is based on their reaction with a 2,4-dinitrobenzenesulfonyl-containing probe showing selective recognition for cysteine, glutathione, and homocysteine [110]. During reaction, the selective cleavage of the 2,4-dinitrobenzenesulfonyl moiety through an aromatic nucleophilic substitution reaction within 10 min leads to the release of *p*-aminophenol (Figure 14). The last one is easily detected by cyclic voltammetry. The linear dynamic range for cysteine, glutathione, and homocysteine is 5–120 μM with detection limits of 1.50, 3.48, and 4.67 μM, respectively. Approach is applicable for the evaluation of total biothiol content in human plasma.

The application of an organic mediator in a solution or in an immobilized form provides the possibility of simultaneous determination of several sulfur-containing antioxidants, especially thiols using discrimination of the electrode reaction kinetics [100,101,102,103,104,105,106,107]. Nevertheless, the problem of sensitive and selective determination of sulfur-containing antioxidants is not resolved. Other mediators, on contrary, show a class selective response that permits measurement of the total contents of thiols in biosamples [109,110].

#### 3.2.4. Polymer-Based Sensors

Electrochemical sensors with polymeric coverages are extensively studied in the analytical chemistry of bioactive compounds [111,112,113,114]. Unfortunately, polymer-based sensors for the determination of sulfur-containing antioxidants have received limited attention. There are several examples of such sensors, which are presented in Table 3.

As one can see from data in Table 3, various types of polymers have been used including conductive [119,122,123] and non-conductive ones [115,116,117,118,120,121]. The last ones are based mainly on the natural phenolic antioxidants [116,120,121]. The molecularly imprinted polymer-based sensors are of high interest as far as providing excellent selectivity in the presence of structurally related compounds [117] and show one of the best analytical characteristics among various sensors discussed above.

**Figure 15:**
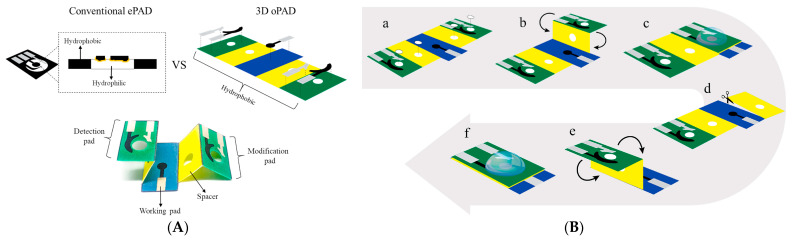
Schematic presentation of the oPAD and polycystene oPAD fabrication. (**A**) The comparison configurations of a conventional ePAD vs. a oPAD and the components of oPAD; (**B**) the oPAD operation and detection of cysteine (punch paper at white area (a), fold paper at modification side onto the top of working pad (b), electropolymerization of cysteine (c), tear the paper at modification side off (d), fold paper at detection side on the top of working pad (e), and electrochemical measurement of cysteine (f)). Reprinted from [118], copyright 2022, with permission from Elsevier.

An interesting novel l-cysteine sensor has been developed using in situ electropolymerization of l-cysteine at the surface of oPAD (origami paper-based analytical device) [118]. oPAD has been created by printing three electrodes on the hydrophobic area of a paper device (Figure 15A).

Then, a spacer layer at the device was constructed to create the solution reservoir. This layer enables the solution to remain on the working electrode surface and aligns three electrodes in the same planar during modification and detection making it easier for the operation. The components of a single oPAD consist of the modification pad, spacers, detection pad, and working pad. The modification and detection pads were separated from each other to prevent cysteine contamination from electropolymerization step to the cysteine detection step. The polycysteine layer was obtained by electropolymerization after dropping 120 μL of 3 mM cysteine in phosphate buffer pH 8.0 onto the device (Figure 15B) and nine-fold potential cycling from −0.6 V to +2.0 V at a scan rate of 100 mV s^–1^. The designed sensor platform allows l-cysteine quantification in the presence of typical bakery product components, in particular thiamine (vitamin B_1_), nicotinic acid (vitamin B_3_), folic acid (vitamin B_9_), ascorbic acid (vitamin C), glucose, fructose, sucrose, Na^+^, Mg^2+^, Fe^2+^, and Ca^2+^ [118]. Other advantages of the novel sensor application are its simplicity, readiness for use, inexpensiveness, portability, and the rapidity of the response to cysteine (the total analysis time is approximately 10 min per sample). The sensing platform could be considered a perspective tool for real-time monitoring of food quality.

#### 3.2.5. Sensors Based on the Combination of Nanomaterials

To improve the analytical characteristics and the selectivity of sulfur-containing antioxidants, the combination of various types of the electrode surface modifiers is widely applied. Layer-by-layer modification or drop casting of preliminary synthesized nanocomposites and hybrids suspended or dispersed in suitable solvents are usually used. The sensors based on such combinations are summarized in Table 4.

Sensors based on the combination of various types of nanomodifiers show improved analytical characteristics for sulfur-containing antioxidants. The detection limits achieved the nM-pM level [134,138,139,146,150,152,154,156,157,158,160,161]. The reason for this is the synergetic effects of different modifiers, in particular:

- High surface area and conductivity due to the carbon-based nanomaterials including 3D porous structures (hierarchical amorphous carbon network [128], MOF [156], MXene [158]);
- Conductivity and adsorption affinity due to the metal NPs and ions [126,127,128,129,131,134,136,140,143,153,158,161,166] as well as electrochemical mediator properties;
- High surface area and electrochemical mediator function due to the metal oxide NPs and their composites [124,125,136,138,141,151,157,165];
- Anti-fouling effects due to the presence of polymers like Nafion [129], chitosan [148,163] or polyethylene glycol [163];
- High affinity of polymers including molecularly imprinted ones to the sulfur-containing antioxidants [141,142,143,144,145,146,147,148,149,156,157].

Furthermore, the number of sensors for the homocysteine determination with satisfactory sensitivity and selectivity is increased in comparison to the sensors based on the single modifier.

**Figure 16:**
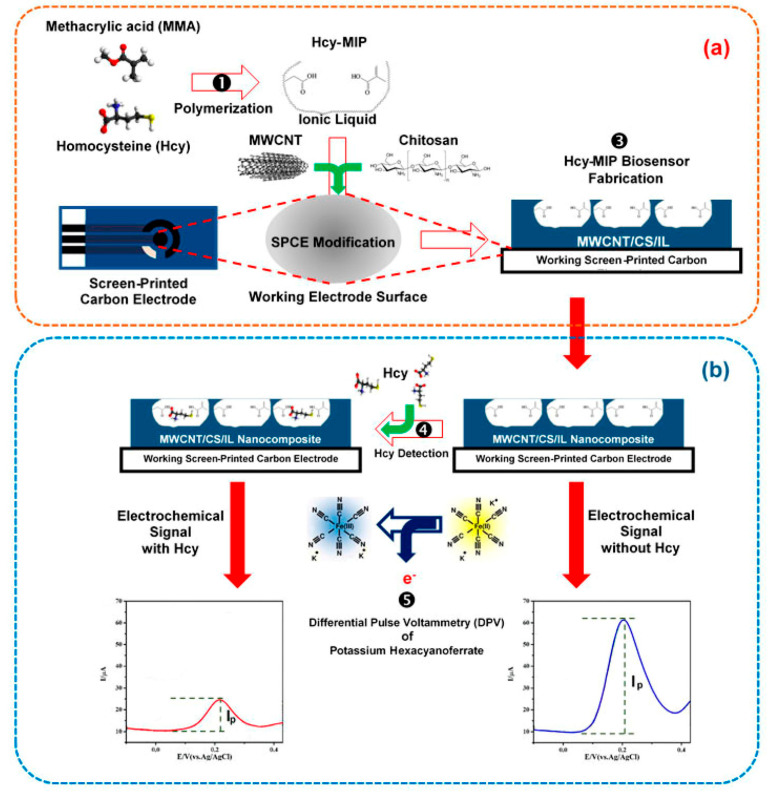
Scheme for homocysteine (Hcy) determination with the MIP electrochemical sensing platform. (**a**) The screen-printed carbon electrode (SPCE) was modified with the mixture of Hcy-MIP and carbon nanotube/chitosan/ionic liquid (CNT/CS/IL) nanocomposite; (**b**) Hcy detection by measuring the differential pulse voltammetric response using [Fe(CN)_6_]^3−/4−^ as an electroactive probe molecule. Reprinted from [148]; published under Creative Commons CC BY license.

The sensors based on the multi-modifier application exhibit much better selectivity of the response to target sulfur-containing antioxidants in the presence of the most typical components of biological samples like human plasma, serum, and urine. The absence of the interfering effect from other S-containing amino acids and glutathione is a key achievement. One of the approaches to provide specific recognition of the analytes is fabrication of molecularly imprinted polymers (MIP). MIP-based sensors allow obtaining of a highly sensitive response to homocysteine [148,149] and l-cysteine [152]. A label-free electrochemical sensor for reliable homocysteine detection has been fabricated from screen-printed carbon electrode covered with carbon nanotube/chitosan/ionic liquid nanocomposite with MIP synthetized from the methacrylic acid in the presence of trimethylolpropane trimethacrylate as a cross-linking agent and 2,2′-azobis(2-methylpropio nitrile) as a radical-induced polymerization initiator (Figure 16). Differential pulse voltammetry in the presence of ferri-/ferrocyanides mixture has been used for the detection of homocysteine binding to MIP.

An MIP film obtained by electrodeposition of chitosan in the presence of cysteine at the surface of GCE modified with layer-by-layer electrodeposited nanoporous gold and Cu NPs demonstrates an excellent recognition of cysteine [152]. Aptamer-based voltammetric sensors for l-homocysteine are another way to obtain a high selectivity for determination [160,161]. The use of Au NPs as support for thiol aptamer self-assembly provides high affinity and easy immobilization of the aptamer at the electrode surface.

**Figure 17:**
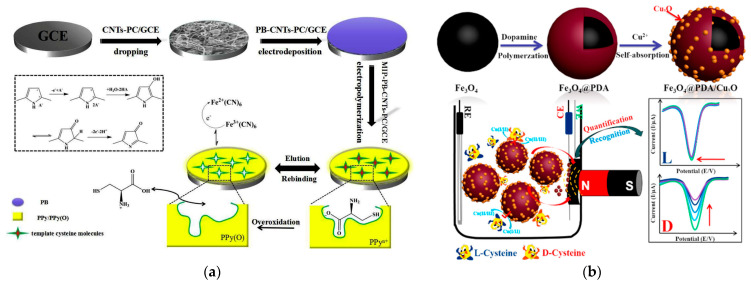
Electrochemical sensors for the selective chiral recognition of cysteine: (**a**) Schematic illustration of fabrication of GCE/MWCNTs–MOF-5-derived porous carbon–Prussian blue nanocube hybrid/Polypyrrole-based MIP sensor for chiral analysis of cysteine enantiomers. Reprinted from [156], copyright 2019, with permission from Elsevier. (**b**) fabrication of the sensor based on Fe_3_O_4_@Polydopamine/Cu_x_O and its response for the highly selective determination of d-cysteine. Reprinted from [157], copyright 2018, with permission from American Chemical Society.

One of the important aspects in the electroanalysis of sulfur-containing amino acids is the possibility of chiral selection of d- and l-isomers that has been successfully achieved using electrodes modified with polymers in combination with redox mediators [156,157] and chiral cross-linked cyclodextrin MOF–Pt NPs [166]. An electrode based on the three-dimensional conductive MIP deposited at the MWCNTs–MOF-5-derived porous carbon–Prussian blue nanocube hybrid immobilized at the GCE shows mediated amplification for chiral analysis of cysteine enantiomers (Figure 17a) [156]. MIP has been obtained by polypyrrole electropolymerization in the presence of d- and l-cysteine. The template elution has been carried out through a simple overoxidation/dedoping process.

An original voltammetric sensor has been created for the selective and ultrasensitive determination of d-cysteine using magnetic nanoparticles covered with polydopamine and self-adsorbed Cu(II) ions forming Cu_x_O [157] (Figure 17b). In the presence of d-cysteine, a stable d-Cys-Cu^2+^-d-Cys complex is formed and proportional decrease in the reduction current of Cu^2+^indicator under conditions of square wave voltammetry is registered. The binding affinity of l-Cys is significantly less and almost no changes in the voltammetric response were observed. Recognition of methionine enantiomers has been achieved using GCE modified with a platinum NP-modified chiral cross-linked cyclodextrin MOF [166]. γ-Cyclodextrin used for MOF synthesis possesses numerous chiral sites and a larger active area. The hydrophobic cavity can selectively bind methionine chiral molecules and form diastereomeric complexes to achieve the effect of chiral recognition. Unfortunately, the oxidation potentials of both l- and d-methionine are close to each other when an oxidation current ratio of 1.98 is achieved. Similar recognition by the difference in the oxidation currents has been obtained for l- and l-cysteine using sensors based on gold electrodes modified with polyarylenephthalides [167].

Thus, electrochemical sensors based on a combination of various types of modifiers are effective and promising tools for the sensitive and selective quantification of sulfur-containing antioxidants. Nevertheless, their simultaneous determination is of high importance and cannot be performed using the electrochemical sensors mentioned above due to the close oxidation potentials or insufficient sensitivity towards all sulfur-containing antioxidants. This problem can be solved by combining liquid chromatography with electrochemical detection.

## 4. Simultaneous Determination of S-Containing Antioxidants by Liquid Chromatography with Electrochemical Detection

Electrochemical sensors can be successfully combined with liquid chromatography to perform simultaneous quantification of sulfur-containing antioxidants, in particular S-containing amino acids and glutathione (Table 5). Electrochemical detection can be realized using coulometric or amperometric modes.

High sensitivity, low detection limits, lower background currents, and less potential interferences, as well as absolute quantification excluding the use of calibration graphs and the possibility of trace analysis make coulometric detection universal and perspective from practical point of view [180]. Several electrodes in series (up to sixteen) can be used on the principles of coulometric array [181]. The application of the potential on the first electrode which is 0.2–0.3 V lower than the potential of the detection electrode provides removal of interfering and undesired oxidizable components and elimination of their effect on the analytical signal. The porous carbon electrodes with large active surfaces are usually applied in coulometric detectors [182].

Amperometric detection is based on the combination of flow-cell with the standard electrodes usually used in voltammetry. Screen-printed electrodes allow miniaturizing of the system and are widely applied nowadays, which is also stipulated by producers of commercial electrodes and cells. The slow kinetics of sulfur-containing antioxidants oxidation at bare electrodes significantly decreases the sensitivity and requires the application of high positive working potentials. A promising approach to solve this problem is the application of chemically modified electrodes based on Au nanoparticles [177], Ag microparticles [178] or carbon nanomaterials [179].

**Figure 18:**
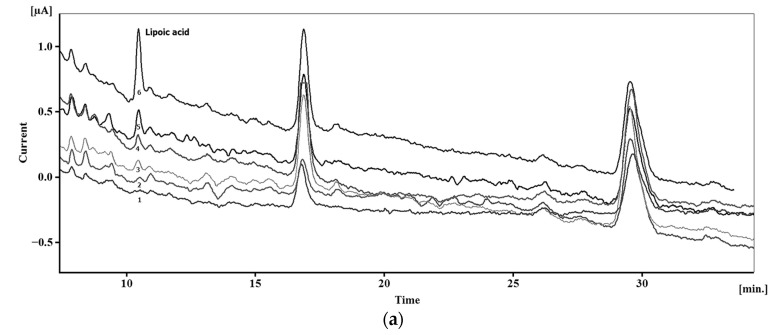

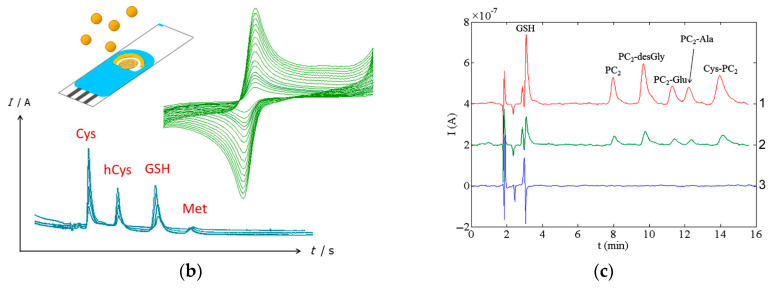
Typical chromatograms with electrochemical detection of sulfur-containing antioxidants: (**a**) Chromatograms of plasma without addition of lipoic acid (**1**) and with additions of 1 (**2**), 5 (**3**), 7.5 (**4**), 15 (**5**) and 50 nM (**6**) of lipoic acid. Reprinted from [169], copyright 2017, with permission from John Wiley & Sons, Ltd. (**b**) Schematic illustration of the simultaneous determination of cysteine (Cys), homocysteine (hCys), glutathione (GSH), and methionine (Met) using green-synthesized Au nanoparticles/Au SPE. Reprinted from [177], copyright 2019, with permission from Elsevier. (**c**) Chromatograms of glutathione (GSH), phytochelatin 2 (PC_2_) and its isoforms at a concentration of 0.10 mM (**1**), 30 μM (**2**), and the corresponding chromatogram of the blank (**3**) using amperometric detection at a commercial bare SPCE (applied potential of 1.0 V). Reprinted from [179], copyright 2015, with permission from Elsevier.

Typical chromatograms with electrochemical detection of sulfur-containing antioxidants are presented in Figure 18 and exhibit well-resolved peaks.

**Figure 19:**
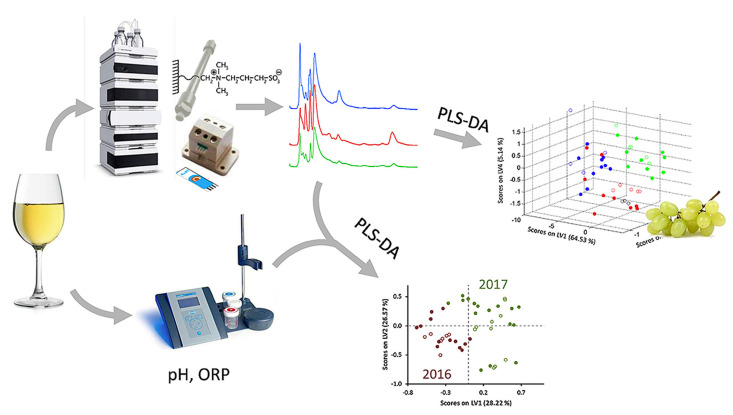
Discrimination of white wines from harvest year and varieties based on hydrophilic interaction liquid chromatography with amperometric detection, pH and redox potential data. Red, blue and light green lines and dots correspond to various grape varieties. Reprinted from [176], copyright 2019, with permission from Elsevier.

Further interesting data have been obtained by coupling the high-performance liquid chromatography data with partial least squares discriminant analysis [176]. Aminothiols content as biomarkers allows for the discrimination of white wines in terms of harvest year. Chromatographic peaks area of thiols, pH of wines, and their redox potentials have been used for the successful discrimination of wines from different harvest years with a global classification rate of 97.8% (Figure 19). Moreover, the high-performance liquid chromatography with amperometric detection at Au SPE exhibits characteristic fingerprints to be applied for wine classification according to three wine varieties using chemometric treatment.

## 5. Conclusions and Future Development

Sulfur-containing antioxidants are important biomarkers to be controlled for diagnosis purposes. Both high and low levels of S-containing amino acids and glutathione lead to the development of various pathologies in human organism. The presence of thiol, disulfide, or methylthiol groups in the structure of sulfur-containing antioxidants makes them electroactive and able to be measured using electrochemical methods.

Many electrochemical sensors have been developed for sulfur-containing antioxidants quantification. Most of them are focused on l-cysteine determination while homocysteine, among others, is less studied. The absence of an electrochemical response in the available potential range or high oxidation potentials are observed for sulfur-containing antioxidants at the traditional bare carbon-based and platinum electrode. To solve this problem, a great number of chemically modified electrodes have been fabricated using carbon nanomaterials, metal and metal oxide nanostructures, polymeric coverages, inorganic and organic redox mediators, including nanosized ones, as well as various types of nanocomposites and combinations of nanostructured modifiers mentioned above. Chemical or electrochemical synthesis of nanomaterials and hybrid structures are usually used. Chemical methods are time-consuming (from several hours up to several days); require the application of various additional reagents including toxic reducing/oxidizing agents and organic solvents; include several stages of various treatment cleaning, and drying of the material obtained; and need rigorous control of the chemical composition and structure after each step. The following electrode surface modification can also take several steps as well as a special form of modifier immobilization in some cases. Electrochemical approaches for nanosized material production occur directly at the electrode surface, require the minimum number and quantity of reagents, and are eco-friendly and fast (the procedure usually takes several minutes). The properties and quantity of modifier deposited are easily controlled by variation in the electrolysis parameters applied, which provides high reproducibility of the modifier layer and, as a result, stability of the sulfur-containing antioxidants’ response.

The best analytical characteristics of sulfur-containing antioxidants and perfect selectivity of their response including possibility of chiral recognition and quantification have been demonstrated using sensors based on hybrid nanocomposites. The search and enlargement of the modifiers for sensor creation providing fully resolved redox signals of S-containing amino acids enantiomers are of high practical importance and need to be further investigated. Probably, the modifiers with inherent chirality to be synthesized for each analyte using preliminary molecular docking studies in order to obtain coverage demonstrating an unprecedented ability to provide separate voltammetric peaks for the enantiomers.

Another interesting trend in the future development of the field is the use of mathematical design methods for optimization of the conditions of the nanomaterials and hybrid composite syntheses, which can significantly simplify the procedure and improve fabrication process. In the case of molecular imprinting, application of theoretical and computational approaches can give a rational design and synthesis of MIP with predetermined recognition properties.

Electrode surface fouling is one of the problems to be solved in the electroanalysis of sulfur-containing antioxidants. The renewal of the electrode surface takes time and increases the consumption of the modifiers. The application of polymers (Nafion, polyethylene glycol, chitosan) prevents electrode surface passivation and partially solves this problem. The most effective way is the creation of sensors with anti-fouling properties that can be used for several measurements or sensors whose surface can be regenerated by easy washing of the surface or by electrochemical treatment. Another approach to overcome electrode surface fouling is its application in flow conditions. The effectivity of the combination of liquid chromatography with electrochemical detection has been confirmed by the example of all sulfur-containing antioxidants under consideration. Various types of flow-injection analysis could also be very useful due to the continuous cleaning of the electrode surface from oxidation products and sample components by carries stream. Nevertheless, there is lack of attention to flow methods in electroanalysis of sulfur-containing antioxidants nowadays.

As for the real samples analysis, attention should be paid to the selective and simultaneous determination of sulfur-containing antioxidants at the physiological concentrations and abnormal decreased/increased levels. Currently, in the majority of studies (especially in the case of homocysteine), the spiked analysis has been performed while the level of target sulfur-containing antioxidant cannot be measured. Furthermore, the 24 h urine sample has to be used for correct data interpretation. Applications to clinical samples are also missed.

Summarizing the above, development of novel electrochemical sensors for quantification of sulfur-containing antioxidants with improved analytical and operation characteristics are still required and will be in focus in the nearest future.

## Figures and Tables

**Table 1:** Electrochemical sensors based on carbon nanomaterials for sulfur-containing antioxidants and their analytical capabilities.

Analyte	Sensor	Detection Mode	Supporting Electrolyte	Detection Limit (μM)	Linear Dynamic Range (μM)	Samples Studied	Ref.
l-methionine	Acid treated MWCNTs/GCE	LSV	0.1 M H_2_SO_4_	270	360–2600; 3600–6900	Pharmaceutical dosage forms	[36]
	Electrochemically reduced GO/GCE	DPV	PB ^1^ pH 5.5	100	450–4900	—	[47]
	Activated 3D-printed electrodes based on polylactic acid/graphene filament	SWV ^2^	0.10 M PBS ^3^ pH 7.4	1.39	5.0–230; 230–3000	Spiked serum samples	[48]
	Fullerene-C_60_ modified gold electrode	CV	0.1 M KNO_3_ pH 6.0	8.2	Up to 100	Root beer syrup and methionine pills	[49]
l-cysteine	Acid treated MWCNTs/GCE	LSV	0.1 M H_2_SO_4_	64	75–1900	—	[36]
	Electrospun carbon nanofibers CPE ^4^	A ^5^	0.1 M PB pH 7.0	0.1	0.15–64	—	[50]
	Boron-doped carbon nanotube/GCE	A	0.067 M PB pH 7.4	0.26	0.78–200	Spiked NongFu-Spring syrup	[51]
	Ordered Mesoporous Carbon/GCE	A	0.10 M PB pH 2.0	0.0020	18−2500	—	[52]
Glutathione	Acid treated MWCNTs/GCE	LSV	0.1 M H_2_SO_4_	43	76–1800	—	[36]
	MWCNTs/screen-printed electrode	DPV	0.10 M PB pH 7.4	0.47	—	Rat plasma	[53]
α-Lipoic acid	Acid treated MWCNTs/GCE	LSV	0.1 M H_2_SO_4_	19	26–180; 210–780	Pharmaceutical dosage forms	[54]

^1^ Phosphate buffer. ^2^ Square-wave voltammetry. ^3^ Phosphate buffer saline. ^4^ Carbon paste electrode. ^5^ Amperometry.

**Table 2:** Analytical characteristics of sulfur-containing antioxidants at the electrodes based on metal, metal oxide, and other inorganic compound nanostructures.

Sensor	Analyte	Detection Mode	Supporting Electrolyte	Detection Limit (μM)	Linear Dynamic Range (μM)	Samples Studied	Ref.
**Metal nanomaterials**							
Au NPs/Graphite electrode	l-cysteine	CV, cathodic peak	0.1 M NaOH	0.6 × 10^−6^	(1–14) × 10^−6^	—	[55]
Nanoporous Au/GCE		A, +0.65 V	0.2 M PB ^1^ pH 7.0	0.050	1–400	—	[56]
Au NPs/Nafion/GCE		A, +0.45 V +0.35 V	0.1 M PB pH 2.0 0.1 M PB pH 7.0	1.0 1.0	3.0–50 2.0–80	— —	[57]
Bismuth nanostructure-CILE ^1^		SWV	0.1 M acetate buffer pH 4.5	0.5	1–500; 500–2000	Human serum	[58]
Electrodeposited Cu/screen-printed Au electrode		A, +0.5 V	0.1 M NaOH	0.21	1–400; 400–1800	Human and rabbit serum	[59]
Ag NPs/Carbon fiber clustered electrode		CV	PB pH 8.0 with 0.5 M KNO_3_	1.0 × 10^−7^	1.0 × 10^−7^–0.010; 0.010–10,000	Mouse whole blood	[60]
Cu nanowires in poly(dimethyl diallyl ammonium chloride)/GCE		A, +0.2 V	0.1 M NaOH	0.19	2.0–260	Human plasma	[61]
Co NPs/ITO ^2^	Glutathione	A, +0.75 V	0.25 M NaOH	0.005	0.005–0.675; 1–11	Eye drops	[62]
**Bimetallic NPs and nanosheets**							
Ag-Au core–shell bimetallic NPs/GCE	l-methionine	A, +0.63 V	PB pH 7.4	30	50–500	—	[63]
Ru-Pt/Nanoporous Au film electrode		DPV	0.1 M PB pH 7.0	0.002	0.006–0.105; 3–102	Human urine	[64]
Ag–Pd bimetallic NPs/GCE	l-cysteine	—	0.1 M PBS pH 7.0	—	—	—	[65]
BiPr composite nanosheet/GCEs		CV	0.1 M KCl	0.21	1–2000	—	[66]
Ag–Hg/Nafion/Cu	l-cysteine	DPV	Brdička supporting electrolyte	0.088	1–10	Cysteine-rich peptides from tobacco cells	[67]
	Glutathione			0.139	20–100		
Ag–Bi/Nafion/Cu	l-cysteine			0.41	1–10		
	Glutathione			0.244	20–100		
**Metal oxide nanostructures**							
Sb_2_O_3_–CPE ^3^	Glutathione	SWV	0.2 M Britton-Robinson buffer pH 6.0	0.34	2–300	Urine samples	[68]
	Reduced oxidized			0.10	2–400		
1.5 μg NiO NPs/GCE	Glutathione	DPV	50 mM PB pH 7.2	—	200–6000	—	[69]
AgO/PGE ^4^	Methionine	CLSV ^5^	0.1 M NaOH + 17.7 μM AgNO_3_	0.42	60–500	Healthy human serum	[70]
CuO nanoflowers/GCE		DPV	0.1 M PB pH 7.0	0.3	1.0–300	Human urine	[71]
Mn_2_O_3_/SPCE ^6^		DPV	0.1 M PB pH 7.0	0.001	1.0–610	Sea food samples	[72]
CuO nanostructures–CILE	l-cysteine	A, +0.30 V CV	0.2 M PB pH 7.0	— 0.51	10–5000 5–4000	Soya milk	[73]
Co_3_O_4_ NPs/GCE		A, +0.65 V	0.1 M PB pH 11	0.07	0.20–75	Human urine	[74]
Free-standing TiO_2_ nanotube array/GCE		A, +0.75 V	0.1 M PB pH 7.0	100	100–10,000	Human serum	[75]
Electrodeposited Cu_2_O nanostructures/FTO ^7^	l-cysteine	CV	0.5 M Na_2_SO_4_	0.0062	0–5.0	Human urine	[76]
	Homocysteine			0.013	0–5.0		
	Glutathione			0.0275	0–5.0		
SnO_2_ NPs in cetyltriphenylphosphonium bromide/GCE	α-Lipoic acid	DPV	Britton–Robinson buffer pH 4.5	0.13	0.50–50; 50–400	Pharmaceutical dosage forms	[77]
CeO_2_·Fe_2_O_3_ NPs/GCE		DPV	0.1 M PB pH 7.0	0.053	0.075–7.5; 7.5–100	Pharmaceutical dosage forms	[78]
Heterostructure CeO_2_-CuO nanocomposite/GCE	l-cysteine	A, +0.80 V	0.1 M PB pH 7.0	10	10–5000	Pond water	[79]
CeO_2_-SnO_2_ nanocomposite/GCE		A, +0.80 V	0.1 M PB pH 7.0	16	16–2000	Pond water	[80]
Heterostructure NiO–SnO_2_ nanocomposite/GCE		A, +0.60 V	0.1 M PB pH 7.0	0.01	10–6000	Pond water	[81]
Elbow-shaped Sb-doped ZnO nanowires/GCE		A, +0.50 V	0.1 M PB pH 6.9	0.025	0.075–100	Human serum and acetylcysteine tablets	[82]
Electrodeposited Co-Gd_2_O_3_ nanocomposite/GCE		A, +0.40 V	0.1 M NaOH	0.23	1–100	Milk and cysteine capsules	[83]
CuO@Si core–shell nanoparticles/GCE		DPV A, +0.90 V	0.1 M PB pH 7.0	— 0.015	20–240 1.25–100	—	[84]
**Nanomaterials of metal-containing compounds**							
Ce^3+^ doped M-type SrFe_12_O_19_/GCE	l-cysteine	CV	0.1 M PB pH 7.0	0.166	0.5–1.2	—	[85]
Ce-doped Mg–Al layered double hydroxide/GCE		A, + 0.65 V	0.1 M PB pH 7.4	4.2	10–5400	NongFu-Spring syrup	[86]
Co(II)/Al(III) layered double hydroxide/PGE		DPV	0.1 M NaOH	0.1 × 10^−6^	0.1 × 10^−6^–100	Acetylcysteine ablets	[87]
Au NPs–Cu@Cu_x_O nanowires 3D foam hybrid		A, +0.6 V	0.1 M PB pH 7.4	1.25	1.25–1940	Human serum	[88]
Ag doped jalpaite (Ag_3_CuS_2_) pressed electrode		A, +0.04 V	0.1 M PBS pH 7.0	0.024	1–100	Dietary supplements	[89]
Bi_2_Te_2_O_7_ nanospheres/GCE		CV	0.1 M KCl	0.046	0.1–2000	—	[90]
Bi nickelate nanorods/GCE		CV	0.1 M KCl	0.087	0.5–2000	—	[91]
In situ electrodeposited Ag NPs/NiFe contained Prussian blue analog/GCE	Glutathione	DPV	0.1 M PB pH 6.5 in the presence of 40.0 mM Cl¯	0.12 × 10^−6^	0.5 × 10^−6^–5.0	Serum, white grape, and vegetables	[92]
ZnS/ZnAl_2_S_4_ nanocomposite-CPE	l-methionine	SWV	0.1 M PB pH 7.0	0.010	0.05–5.0; 5.0–800	Human urine and serum	[93]

^1^ Carbon ionic liquid electrode. ^2^ Indium tin oxide electrode. ^3^ Carbon paste electrode. ^4^ Pencil graphite electrode. ^5^ Cathodic linear sweep voltammetry. ^6^ Screen-printed carbon electrode. ^7^ Fluorine-doped tin oxide glass electrode.

**Table 3:** Polymer-based electrochemical sensors for sulfur-containing antioxidants and their analytical characteristics.

Analyte	Sensor	Detection Mode	Detection Limit (μM)	Linear Dynamic Range (μM)	Samples Studied	Ref.
α-Lipoic acid	5% Chitosan-polyurethane membrane modified gold electrode	DPV	4.931	5–200	Synthetic urine	[115]
	Poly(vanillin)/Pt electrode	AdASWV ^1^	25	30–2000	Synthetic urine	[116]
l-cysteine	Methacrylic acid based MIP ^2^ modified carbon paste electrode	DPV	0.0096	0.02–0.18	Tap water and human plasma	[117]
	Polycysteine/all-in-one origami paper-based analytical device	DPV	5.5	10–800	Wheat flour, bread, and cake	[118]
	Poly(caffeic acid)/GCE	CV	99	100–5000	—	[119]
	Polyeugenol/GCE	CV	—	—	—	[120]
	Polyeugenol/Ti electrode	CV	—	1–100	—	[121]
Glutathione	Poly(caffeic acid)/GCE	CV	2.2	100–5000	—	[119]
l-methionine	Electropolymerized film of 1,8,15,22-tetraaminophthalocyanato-copper(II)/GCE	DPV	0.027	50–500	Spiked human serum	[122]
	Electropolymerized film of 3-amino-5-mercapto-1,2,4-triazole/GCE	A, +1.35 V	4.12 × 10^−4^	0.10–100	Human urine	[123]

^1^ Adsorptive anodic square wave voltammetry. ^2^ Molecularly imprinted polymer.

**Table 4:** Electrochemical sensors based on a combination of various modifiers and their capabilities in sulfur-containing antioxidants analysis.

Sensor (from Inner to Outer Layer)	Analyte	Detection Mode	Detection Limit (μM)	Linear Dynamic Range (μM)	Samples Studied	Ref.
**Carbon nanomaterials with metal-containing nanostructures**						
Screen-printed graphene electrode/MnO_2_	α-Lipoic acid	SWASV ^1^	0.42	1.4–20	Dietary supplement tablets	[124]
Carbon paste electrode—single-walled carbon nanotubes–Co_3_O_4_ NPs		SWV	0.37	2–100	Dietary supplements	[125]
GCE/Acetylene black–dihexadecyl phosphate/Electrodeposited Au NPs	l-homocysteine	A, +0.6 V	0.6	3.0–1000	Human serum	[126]
GCE/rGO ^2^–Au NPs		A, +0.2 V	6.9	10–80	—	[127]
GCE/3D pothole-rich hierarchical amorphous carbon network-encapsulated Ni NPs	l-cysteine	CV	0.15	0.8–85	Human serum and urine	[128]
GCE/Nanohybrid rGO-Nafion@Pd NPs		A, +0.6 V	0.15	0.5–10	Human urine	[129]
GCE/Chitosan/1D caterpillar-like MnO_2_–carbon nanocomposite		CA ^3^, +0.5 V	0.022	0.5–680	—	[130]
GCE/MWCNTs/Ru NPs		DPV	0.353	0–200	Acetylcysteine tablets	[131]
GCE/Carbon nanotube-supported Ga		DPV	0.05	0–200		[132]
GCE/Hierarchical 3Dtraw-bundle-like Mn-La oxides–rGO nanocomposite		A, +0.6 V	0.1	0.5–32.5; 32.5–832.5	Serum and commercial syrup	[133]
GCE/Ni_2_P nanocrystallines anchored on black phosphorus nanosheets		A, +0.3 V	0.002	0.02−20; 20−100; 100−1000	Human serum and urine	[134]
GCE/p-type MoS_2_ nanocube modified poly(diallyl dimethyl ammonium chloride)-mesoporous carbon composites		A, +0.65 V	0.090	0.45–155	Human urine	[135]
Activated 3D printed graphene electrode/Anchored Prussian blue NPs		A, +1.1 V	0.858	3.0−230	Spiked human serum	[136]
GCE/CuFe_2_O_4_–rGO–Au NPs composite		CV	0.383	50−200	Human urine	[137]
*N*-doped porous carbon nano-polyhedron embedded with Co_3_O_4_		A, +0.10 V	0.0069	0.05–5200	Human serum	[138]
GCE/core–shell CNTs@NiAl layered double hydroxide nanoarchitectures		A, +0.19 V	0.003	0.05–780	PC-12, MCF-7, MDA-MB-231 cells, serum, and urine	[139]
GCE/Graphene quantum dots/Electrodeposited Ag NPs	Glutathione	DPV	0.08	0.10–200	—	[140]
**Combined coverages with polymers**						
GCE/Carboxylated MWCNTs–polyindole/Ti_2_O_3_	α-Lipoic acid	AdADPV ^4^	0.012	0.39–115.8	Tomatoes and broccoli	[141]
GCE/polyaniline–zinc bismuthate nanocomposite	l-Cysteine	CV	0.012	0.05–2000	—	[142]
GCE/Poly(Trypan Blue)/Elecrodeposited AuNPs		DPV	0.006	5.0–270.0	Human blood and urine	[143]
GCE/MWCNTs/Poly(*p*-coumaric acid)		DPV	1.1	7.5–50; 50–1000	Human urine	[144]
GCE/Highly conductive carbon black/Poly(syringic acid)		CA, +0.04 V	0.639	20–100; 100–1000	Simulated blood serum and chicken samples	[145]
GCE/Polythionine/Cu	Glutathione	CV	0.0039	0.01–1000	Injection sample and human urine	[146]
GCE/CNTs/Poly(caffeic acid)		CV	0.5	50–5000	—	[147]
SPCE ^5^/MWCNTs–Chitosan–Ionic liquid–Methacrylic acid based MIP	Homocysteine	DPV	1.2	50–150	Human serum	[148]
Magnetic graphite epoxy composite/Magnetic MIP based on 2-hydroxyethyl methacrylate and trimethylolpropane trimethacrylate		DPV	0.030	0.1–2; 4–70	Real urine, synthetic plasma and urine	[149]
**Other combinations of nanomaterials**						
GCE/Nb_2_CT*_x_* MXene@Pt nanocomposite	l-cysteine	DPV	4.1 × 10^−5^	0.001–10	—	[150]
Carbon paste electrode–Ionic liquid–benzoylferrocene–-MWCNTs decorated with Co_3_O_4_ NPs		DPV	0.04	0.1–100	Urine and water samples	[151]
GCE/Nanoporous Au/Cu NPs/Chtisan-based MIP		DPV	7 × 10^−5^	5 × 10^−4^–10	Bovine serum and sauce of instant noodle	[152]
GCE/Electrochemically reduced graphene oxide@1,10-phenanthroline-5,6-dione-Cu^2+^		A, +0.13 V	2.0	10–32,344	Human urine	[153]
GCE/Functionalized MWCNTs–6-amino-4-(3,4-dihydroxyphenyl)-3-methyl-1,4-dihydropyrano [2,3-c]pyrazole-5-carbonitrile		DPV	1.6 × 10^−5^	7 × 10^−4^–200	Human serum	[154]
Carbon paste electrode–graphene–ethyl 2-(4-ferrocenyl-[1,2,3]triazol-1-yl) acetate		DPV	0.9	4.0–2300	Human urine and serum	[155]
GCE/MWCNTs–MOF-5 derived porous carbon–Prussian blue nanocubes hybrid/Polypyrrole-based MIP	l-cysteine	DPV	6.0 × 10^−9^	1.0 × 10^−7^–0.1	Human serum	[156]
	d-cysteine			1.0 × 10^−7^–0.1		
GCE/Fe_3_O_4_@Polydopamine/Cu_x_O	d-cysteine	SWV	8.3 × 10^−5^	0.01–500	Spiked serum	[157]
GCE/MXene–BSA–Au nanoclusters	l-Homocysteine	A, +0.85 V	1.76 × 10^−3^	5.29 × 10^−3^–0.608; 0.608–103	Human serum and cell lysate	[158]
SPE/Graphene oxide functionalized by dopamine		DPV	0.15	0.5–900	—	[159]
GCE/Au NPs/Aptamer		DPV	0.009	0.05–20.0	Human serum and urine	[160]
GCE/Graphene/Au NPs/Aptamer		DPV	0.005	0.05–20.0	Human serum	[161]
GCE/Amino group electrochemical grafting from ammonium carbamate/Electrodeposited Au@Pt NPs	Glutathione	A, 0.435 V	0.051	0.1–11	Spiked human serum	[162]
GCE/Cu(II) –porous magnesium metasilicate–Ag NPs–polyethylene glycol–Chitosan		SWV	0.03	0.1–125	Dietary supplements and human serum	[163]
Carbon-containing electrode/MWCNTs/Vitamin B_12_	l-methionine	DPASV ^6^	0.12	0.1–5.0	Pharmaceutical dosage forms	[164]
Carbon paste electrode–1-Butyl-3-methylimidazolium hexafluorophosphate–ethyl 2-(4-ferrocenyl[1,2,3]triazol-1-yl) acetate–CeO_2_–ZnO nanocomposite		DPV	0.015	0.05–600	Spiked human serum and urine	[165]
GCE/Chiral cross-linked cyclodextrin MOF–Pt NPs	l-methionine	DPV	0.33	2–200	Human serum	[166]
	d-methionine		0.60	2–200		

^1^ Square-wave anodic stripping voltammetry. ^2^ Reduced graphene oxide. ^3^ Chronoamperometry. ^4^ Adsorptive anodic differential pulse voltammetry. ^5^ Screen-printed carbon electrode. ^6^ Differential pulse anodic stripping voltammetry.

**Table 5:** Figures of merit of liquid chromatography with electrochemical detection in quantification of sulfur-containing antioxidants.

Detection	Analyte	Working Electrode	Potential, V	Detection Limit (μM)	Linear Dynamic Range (μM)	Sample	Ref.
Coulometric detector	α-Lipoic acid	2 PGE ^1^	+0.3–+0.7	0.024	—	Dietary supplements	[168]
		2 PGE	+0.45; +0.59	0.00185	—	Human plasma	[169]
	Glutathione	4 PGE	+0.25–+0.80	15 × 10^−9^	—	Cultured hepatocytes	[170]
		2 PGE	+0.75; +0.95	2.1	—	Human blood	[171]
Amperometric detection	Cysteine	Au SPE ^2^	+0.9	12	40–600	Human plasma	[172]
	Cysteinyl-glycine			1	3–30		
	Homocysteine			1	2–30		
	Methionine			0.2	0.5–10		
	Glutathione			1	2–30		
	Cysteine	Ag disk electrode	+0.08	0.2	0.2–20	Bottled white wines	[173]
	Homocysteine			0.3	0.2–20		
	Glutathione			0.2	0.2–20		
	*N*-acetylcysteine			0.3	0.2–20		
	Cysteine	GCE	+0.9	0.005	0.41–8.3	Human plasma and erythrocytes	[174]
	Cystine			0.05	4.16–83		
	Homocysteine			0.003	0.74–14.8		
	Cysteine	Au	+0.85	4.5 × 10^−10^	0.0017–83	Human plasma and erythrocytes	[175]
	Homocysteine			3.7 × 10^−10^	0.0015–74		
	Methionine			4.4 × 10^−10^	0.0013–67		
	Glutathione			2.6 × 10^−10^	0.00065–33		
	Cysteine	Au SPE	+1.0	0.97	3.22–50.0	White wine	[176]
	Homocysteine			0.52	1.74–50.6		
	Methionine			1.26	4.19–50.1		
	Glutathione			0.84	2.81–50.6		
	Cysteine	Green-synthesized Au nanoparticles/Au SPE	+0.9	3.1	20–80	Commercial dietary supplements	[177]
	Homocysteine			0.6	5–30		
	Methionine			1.0	2–10		
	Glutathione			0.1	5–30		
	Cysteine	Ag microparticles modified carbon paste electrode	+0.08	3.8	10–100	Proteins	[178]
	Glutathione			3.9	10–100		
	Dithiothreitol			3.4	5–50		
	Glutathione	Carbon SPE	+1.0	4.15	1–1000	Plants *Hordeum vulgare* and *Glycine max*	[179]
		Graphene/Carbon SPE		1.38	1–1000		
		MWCNTs/Carbon SPE		0.37	1–1000		

^1^ Porous graphite electrode. ^2^ Screen-printed electrode.

## Data Availability

Not applicable.
